# Conception and early pregnancy in the mare: lipidomics the unexplored frontier

**DOI:** 10.1530/RAF-21-0104

**Published:** 2022-02-18

**Authors:** Edwina F Lawson, Christopher G Grupen, Mark A Baker, R John Aitken, Aleona Swegen, Charley-Lea Pollard, Zamira Gibb

**Affiliations:** 1 Priority Research Centre for Reproductive Science, University of Newcastle, Callaghan, New South Wales, Australia; 2 Sydney School of Veterinary Science, Faculty of Science, University of Sydney, Camden, New South Wales, Australia; 3 Nuffield Department of Women’s and Reproductive Health, University of Oxford, Oxford, UK

**Keywords:** lipidomics, mare, oocyte, embryo, conception, lipids

## Abstract

Lipids are dynamic biological molecules that play key roles in metabolism, inflammation, cell signalling and structure. They are biologically significant in the physiology of conception and reproduction. Many of the mechanisms surrounding equine conception and the early feto-maternal dialogue are yet to be understood at a biochemical level. Recently, lipidomic technologies have advanced considerably and analytical strategies have been enhanced and diversified. Consequently, in-depth lipidomic exploration now has the potential to reveal new lipid biomarkers and biochemical relationships that improve our understanding of the processes leading to efficient and successful reproduction. This review considers the role of lipids in conception and establishment of pregnancy, providing new insights into the enigmatic pathways governing early reproductive physiology of the mare.

**Lay summary:**

This paper discusses the role that lipids play in the very early stages of pregnancy in the mare. Lipids are microscopic non-soluble molecules that are important components of living cells. The manuscript discusses how lipids influence the reproductive cycle of mares, including ovulation and the detailed biological process of becoming pregnant. It explains how lipids are identified in a laboratory setting with a newly developing technology known as ‘lipodomics’. The technology may lead to a more detailed understanding of how mares become pregnant. The focus of the paper is on mare reproduction, but it also draws on similarities with reproduction in other mammals. Remarkably there are gaps in much of our knowledge about the finer details of pregnancy in the horse, and the paper summarises what we already know about lipids, highlighting areas for further research.

## Introduction

Lipids play multiple key roles in a diverse range of cellular processes during gametogenesis, fertilisation and pregnancy (Koeberle 2016). In the horse, the details of many reproductive pathways remain undefined, and as a result, we are yet to identify the maternal recognition of pregnancy (MRP) signal (Swegen 2021) or to establish a protocol for conventional *in vitro* fertilisation (IVF) (Leemans *et al.* 2016). As a result, intracytoplasmic sperm injection is the only viable method for producing equine embryos* in vitro*, albeit with a substantially lower efficacy than natural breeding. Studies exploring early equine pregnancy have focused on endocrine shifts (Stout & Allen 2001*a*, *b*, 2002, Raeside *et al.* 2004), embryo and endometrial gene expression (Klein *et al.* 2010, Klein & Troedsson 2011*b*, Klein 2016), protein and receptor profiling (Zavy *et al.* 1979, Bazer & Roberts 1983, Suire *et al.* 2001, Scholtz *et al.* 2009, 2014, Hatzel *et al.* 2015, Lawson *et al.* 2018, Smits *et al.* 2018), miRNA characterisation (Grøndahl & Hyttel 1996) and attempts to identify a putative MRP factor secreted by the conceptus (Ohnuma *et al.* 2000, Klein & Troedsson 2011*a*, Swegen *et al.* 2017). While these studies have advanced our collective knowledge, recent technical and analytical advancements in the field of lipidomics (Xu *et al.* 2020), including the development of methods to investigate interactions between lipids with proteins and peptides (Saliba *et al.* 2015), open the possibility of new discoveries in equine reproductive physiology. This review outlines recent advances in the field of lipidomics and discusses how the study of lipids relates to conception in the mare. It covers areas of potential function that may lead to a better understanding of equine reproduction leading to enhanced clinical implications.

## The emerging field of lipidomics

There is as much variety and complexity to the range of biologically relevant lipids as there are biologically relevant proteins, but the challenges in conducting in-depth lipidomic analyses have meant that proteomic studies have been more numerous (Muro *et al.* 2014). However, recent innovations in the lipidomic pipeline, including mass spectrometry (MS) chromatographic separation and data processing techniques, have contributed to a recent increase in lipidomic analyses (Gross & Han 2011, Xu *et al.* 2020). Lipidomic analyses can be categorised as either ‘targeted’ or ‘untargeted’ depending on what information is being sought. Untargeted lipidomic analyses are suited to screening for novel lipid biomarkers and include shotgun-lipidomic analyses, which are often used in medical research. Targeted lipidomic analyses provide a quantitative measure of specific lipid species as well as the structural characterisation of bioactive lipid species which are often in low abundance (Ferreri & Chatgilialoglu 2012). Both targeted and untargeted lipidomic analyses are conducted using MS, due to its high throughput potential and sensitivity (Wei *et al.* 2019). Prior to MS, lipids must be extracted from the biological sample. These extractions are then separated into different lipid classes or by fatty acyl chain length and unsaturation level, using either gas chromatography, liquid chromatography, or by direct infusion, with each method having specific benefits and applications (Wu *et al.* 2014, Han 2016, Koeberle 2016, Xu *et al.* 2020). The field has evolved so rapidly over the past decade that there is a disparity in methodologies and technologies with little standardisation (Liebisch *et al.* 2019). A key challenge is that no explicit quantitative relationship exists between ion intensity and lipid concentration, with ion intensity of a peak being influenced by sample preparation, ionisation efficiency and detector response (Rustam & Reid 2018). For example, the sterol lipids, which have vital biological functions, cannot be detected in regular lipid extracts due to their low ionisation efficiency in MS. This is overcome in practice by using stable isotope-labelled standards, which aid in specific lipid detection. Furthermore, some highly bioactive lipids are not stable and are readily attacked by free radicals to form oxidised lipids, thereby evading detection (Li *et al.* 2019).

Data processing initiatives such as the LIPID MAPS® database (Fahy *et al.* 2009, O’Donnell *et al.* 2020) and the Lipidomics Standards Initiative (Liebisch *et al.* 2019), aim to help address these challenges. Furthermore, accurate individual lipid identification and advances in bioinformatics have improved both the qualitative and quantitative understanding of lipidomic data and the capacity to interpret and make use of complex datasets. The methodical updating and development of such resources have assisted in increasing the depth and breadth of lipidomic studies in recent years (Liebisch *et al.* 2020).

## Lipid functions and classifications

**Figure 1:**
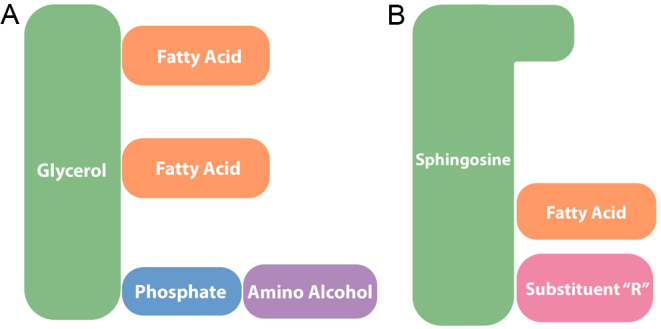
Rudimentary structure of a (A) GP and (B) a SP. Substituent ‘R’ linked to sphingosine will differ based on the molecule, for example, hydrogen (for ceramide), phosphocholine (for sphingomyelin) or sugar (for glycosphingolipid). GP, glycerophospholipids; SP, sphingolipids.

Lipids function predominantly through their interactions with proteins, but many of the pathways by which lipids modulate protein function and structure are not yet fully understood (Saliba *et al.* 2015). Most lipids are combinations of polar head groups with hydrophobic fatty acyl chains that are attached to different lipid backbone structures (Fig. 1). The lipidome arises from the structural diversity that occurs in each lipid due to the variation in the head group, chain length, saturation, branched functional groups, double bond location, cis-trans geometric isomerism and the type of the covalent bond linked to the head group (Xu *et al.* 2020).

**Figure 2:**
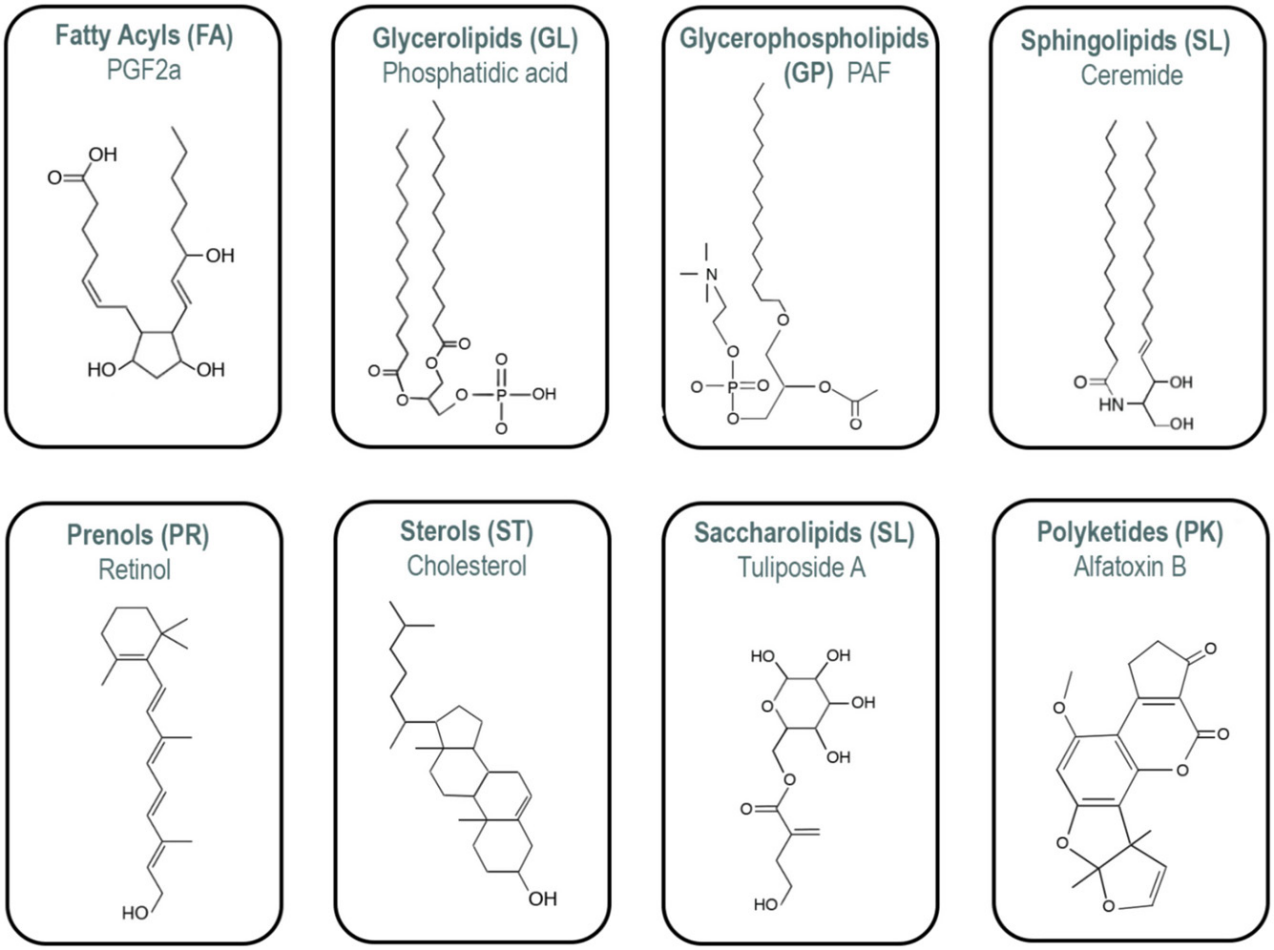
The eight lipid categories with examples of lipid chemical structure. FA, fatty acyls; GL, glycerolipids; GP, glycerophospholipids; PK, polyketides; PR, prenol lipids; SL, saccharolipids; SP, sphingolipids; ST, sterol lipids.

Currently, lipids are categorised according to the LIPID MAPS® classification system and are separated into eight categories: fatty acyls (FA), glycerolipids, glycerophospholipids, sphingolipids, sterol lipids, prenol lipids, saccharolipids and polyketides (Fig. 2). Each category can be further divided into numerous subclasses (Fahy *et al.* 2009). The physiological functions of lipids are wide and varied and they are the main components of biological membranes. Herein, lipids serve as molecular scaffolds that regulate cellular signalling as well as organise and distribute the molecular entities necessary for life processes (Gross & Han 2011). An example of the functional diversity of lipids is the potent glycerophospholipid messenger platelet-activating factor (PAF), a unique pro-inflammatory molecule with immunosuppressive properties (Garrido *et al.* 2017), which is produced when phospholipases enzymatically cleave membrane phospholipids (Shindou *et al.* 2007). Embryos also produce PAF, enhancing vascular permeability, activating local inflammation and instigating alterations in oviductal, endometrial and maternal immune function (O’Neill 2005). On the other hand, the triacylglycerols, which are composed of three fatty acids (FAs) esterified to a glycerol molecule, are primarily involved in the storage of energy. The diversity of the roles of lipids in physiology is a vital feature of the processes surrounding reproduction.

## Lipid hormones are well established in equine reproduction

**Figure 3:**
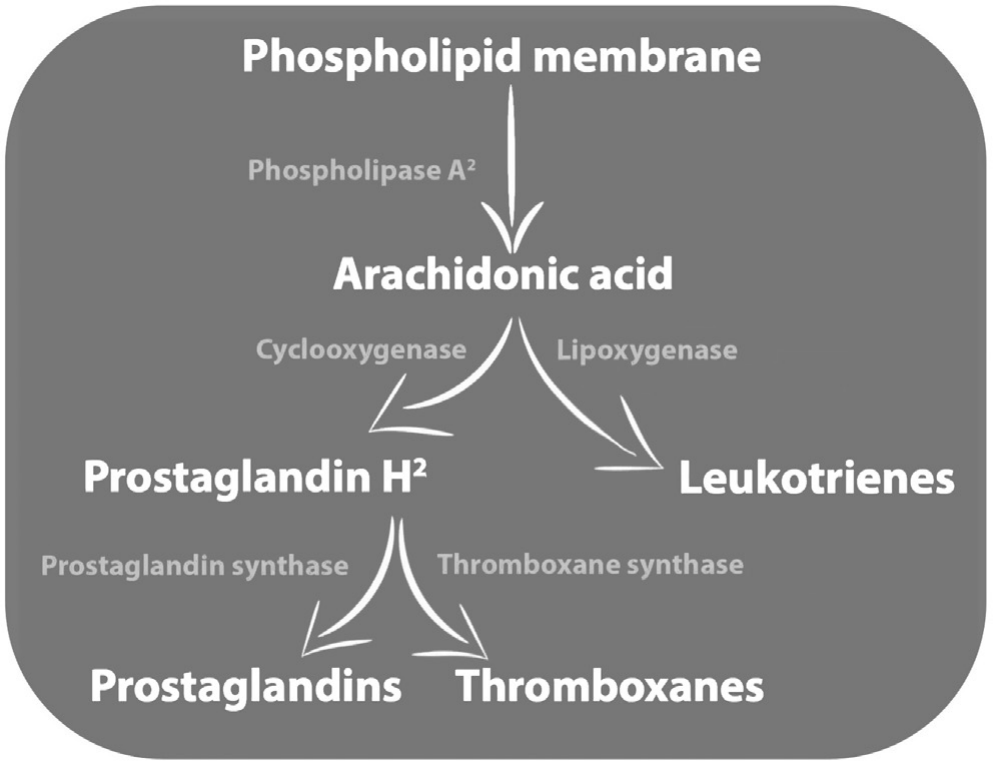
Overview of eicosanoids, including prostaglandins, thromboxanes and leukotrienes produced though arachidonic acid metabolism.

Pregnancy involves a complex hormonal interplay between maternal immunological and neuroendocrine systems in order to sustain the fetus (Shah *et al.* 2019). Reproductive hormones are obvious targets for lipidomic investigations because they regulate reproductive cyclicity and are intrinsically linked to pregnancy. Hence, understanding lipid hormone structure and lipid biosynthesis will lay the groundwork for further investigations into the role of lipids in equine reproduction. Hormones can be broadly separated into three groups based on their chemical structure (Hamid *et al.* 2018): peptide hormones, amino acid hormones and lipid hormones (Norman & Henry 2015). Lipid hormones, which may be further divided into eicosanoids and steroid hormones, are lipid soluble and therefore membrane permeable. Steroid hormones (i.e. oestrogen and progesterone) are derived from cholesterol whereas eicosanoids are derived from plasma membrane FA (Nussey & Whitehead 2001). A diverse group of bioactive lipids, eicosanoids orchestrate inflammation, immunity, oxidative stress and tissue homeostasis (Buczynski *et al.* 2009). Two important groups of eicosanoids are the prostaglandins (PGs) and the leukotrienes (LTs), which play major roles in equine pregnancy. The biosynthesis of PGs and LTs involves the precursor arachidonic acid (AA) which is produced by the phospholipase A2 (PLA2) cleavage of phospholipid membranes (Davies 2008) (Fig. 3).

Production of LTs is initiated by the lipoxygenase arachidonate 5-lipoxygenase [46], and PG production is mediated via the COX enzymes (COX1, COX2) which oxygenate AA to prostaglandin H2. This precursor can then be converted to a range of other prostanoid hormones including prostacyclin and thromboxanes. Most of these lipids are involved in the establishment and maintenance of equine pregnancy, as discussed below.

## Exploration of lipid pathways in equine cyclicity and early pregnancy

### Ovulation

Ovulation involves the rupture of the dominant follicle, with its contents – the oocyte and much of the lipid-rich follicular fluid – being expelled (Avilés *et al.* 2015). In the mare, it appears that much of the follicular fluid is expelled at ovulation into the abdominal cavity (Nambo 2002), but the oocyte appears to accompany the last residuals of fluid into the infundibulum. There is a cascade of biochemical events which lead to ovulation. The mare oestrous cycle is mainly controlled by gonadotropins, which control both ovulation and follicular development. An increased pulse frequency of gonadotropin-releasing hormone from the hypothalamus stimulates luteinizing hormone (LH) release from the pituitary gland (Pinaud *et al.* 1991). This surge in LH is particularly important in setting the events in motion (Samper 2009). However, the mare’s ovulatory events are unique with the LH surge occurring for several days, with levels of LH peaking after ovulation. Nevertheless, the LH level at the time of LH peak is lower than most other species (Yoon 2012).

The low magnitude LH surge further triggers a marked increase in follicular wall PG synthesis just prior to ovulation, with the COX enzyme being a vital rate-limiting step in the biosynthesis of PGs from AA (Sirois *et al.* 2004). Granulosa cells lining the ovarian follicle then synthesise PGF_2α_ and prostaglandin E_2_ (PGE_2_) (Ginther 1992, Sirois & Doré 1997), and the gap junctions that connect these granulosa cells to the cumulus–oocyte complex (COC) break down. Interestingly, it has been shown that in the equine, COX2 gene expression in granulosa cells is a long molecular process, when compared to other species; appearing to be switched on approximately 30 h after the administration hCG for ovulation induction (Boerboom & Sirois 1998). Prostaglandin synthesis, PGF_2α_ and PGE_2_ have a role in follicular wall rupture and can be detected locally in equine follicular fluid (Cuervo-Arango & Martìnez-Bovì 2016); no systemic increase in either of these lipids has been detected at ovulation. As oocyte expulsion must occur for natural fertilisation to take place, the interplay of many lipid hormones, at both local and systemic levels, is essential.

### Contributions of the corpus luteum

The remnants of the ovulated dominant follicle form the basis for the primary corpus luteum (CL); it has a large steroidogenic output, primarily producing progesterone along with high concentrations of AA (Lukaszewska & Hansel 1980). Progesterone could be considered the most important steroid hormone in reproduction and its production by the CL is vital for early pregnancy (Csapo *et al.* 1973, Siiteri *et al.* 1977). The physiological effects of progesterone are facilitated by a receptor-mediated pathway, working as part of a cascade of cyclic events. So for the mare, if there is no embryo present, the CL is lysed, progesterone levels drop and the oestrous cycle begins again. However, if a conceptus is present, it is hypothesised that a yet-to-be identified, anti-luteolytic (Sharp *et al.* 1989) MRP signal is secreted and the CL persists beyond its typical lifespan of 14–16 days (Ginther 1983, Allen 2001, Swegen 2021). Numerous studies have demonstrated that both the equine uterus and the embryo ensure the lifespan and secretory function of the CL during early pregnancy (McDowell *et al.* 1988, Sharp *et al.* 1989, Starbuck *et al.* 1998, Silva *et al.* 2005, Ealy *et al.* 2010). CL persistence is highly variable between species; in ruminants or pigs, like horses, an anti-luteolytic factor is required and in women, a luteotropic factor is required (Aurich & Budik 2015). The luteotropic factor in higher primates is human chorionic gonadotropin (hCG), produced by trophoblast cells and accordingly the hormone used to detect pregnancy in the human. Whilst both women and mares rely on the production of PGF_2α_ for CL lysis, in mares the PGF_2α_ responsible is produced by the endometrium, and in women, it is produced by the ovary (Bennegård *et al.* 1991, Gandolfi *et al.* 1992, Boerboom *et al.* 2004).

Despite indications that bioactive lipids have an important role in CL function (Wiltbank & Ottobre 2003, Hughes *et al.* 2019), little is known about the function of specific luteal lipid mediators in this process, although luteal progesterone is clearly of utmost importance for pregnancy maintenance (Allen 2001). Interestingly, pioneering studies first noted that circulating progesterone in mares, in comparison to many other species, was surprisingly low (Short 1959, Holtan *et al.* 1979) and that 5α-reduced pregnanes including the sterol lipid 5α-dihydroprogesterone (DHP) were surprisingly high (Holtan *et al.* 1991). One study found when ovariectomised mares were supplemented with progesterone, pregnancy could be supported as long as serum concentrations of 2 ng/mL were maintained (Shideler *et al.* 1982). More recently, with the utilisation of liquid chromatography–tandem mass spectrometry (LC-MS/MS), it was found that in the absence of progesterone, DHP stimulated endometrial growth and progesterone-dependent gene expression, maintaining pregnancy as early as the third week (Scholtz *et al.* 2014). This ground-breaking research confirmed DHP as the major progestogen supporting equine pregnancy, validating decades of speculation. Interestingly and again utilising LC-MS/MS technology on serum samples of geldings, cycling and ovariectomised mares, it was demonstrated that equine DHP synthesis is indeed initially dependent on luteal progesterone(Conley *et al.* 2018).

In the cow, there have been reports that lipids ingested through dietary supplementation can alter the luteal response to PGF_2α_ (Plewes *et al.* 2018). A recent study in the dairy cow looked at* in vitro* lipidomic changes in the CL during MRP and suggested that lipids and mRNAs in the CL may regulate a suite of MRP-associated events, including immune cell chemotaxis and cell-cell communication (Hughes *et al.* 2019). Of particular interest was the eicosanoid 15-KETE, which is a major metabolite of AA. In luteal cells, on day 1, a high concentration of 15-KETE induced progesterone production in the presence of LH, but after 7 days, a low concentration of 15-KETE reduced the ability of PGF_2α_ to inhibit LH-stimulated progesterone production. In cattle, the decline in 15-KETE during early pregnancy has been proposed to mediate an increased luteal resistance to PGF_2α_ (Hughes *et al.* 2019). Inspiration for better understanding equine biophysiology can be drawn from such studies. Luteal function and associated lipid mediators could influence equine reproductive cycling and pregnancy outcome, as such further studies of equine CL are certainly warranted.

### Lipids in the oocyte

In the mammalian oocyte intracellular lipids are stored primarily in the form of intracytoplasmic lipid droplets, which contain varying concentrations of triglycerides, phospholipids, cholesterol, free fatty acids (FFA) and proteins (Sturmey *et al.* 2009, Romek *et al.* 2011). It is generally believed the primary purpose of lipid droplets is to provide a rich energy source, supporting events from fertilisation to pre-implantation embryo development (Prates *et al.* 2014). In the oocyte, the energy is most likely generated via mitochondrial FA oxidation (de Andrade Melo-Sterza & Poehland 2021). Fatty acid oxidation can generate roughly 3.5 times more ATP molecules than glucose, and as such, it is an efficient source of energy (Dunning *et al.* 2010). In addition to serving as an energy source, lipids also play a vital role in determining the physical properties of the phospholipid plasma membrane (Amstislavsky *et al.* 2019). However, during maturation lipid stores decrease, and it has been shown that in pig oocytes, triglycerides, phospholipids and cholesterol in intracytoplasmic lipid droplets significantly decrease (by 18, 26 and 24%, respectively) as the oocyte matures and embryonic development progresses (Romek *et al.* 2011).

**Figure 4:**
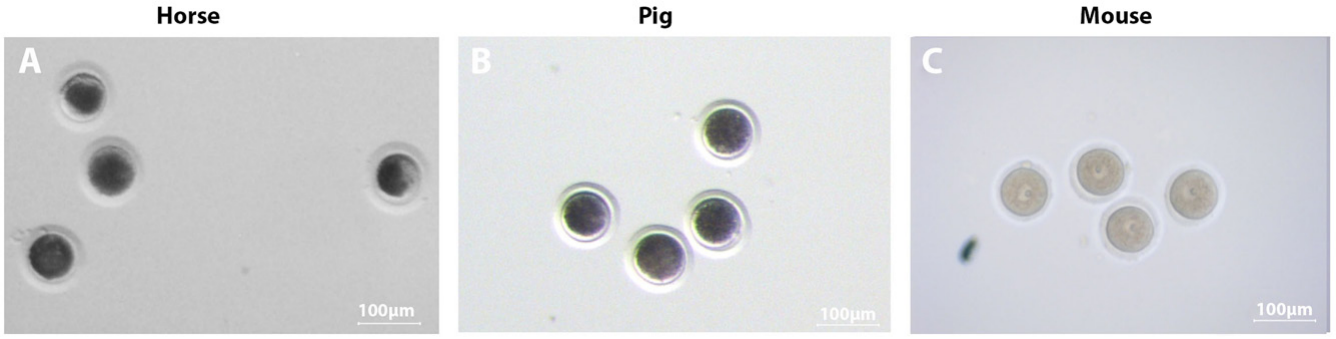
Species-specific differences in oocyte lipid content can be observed in denuded mature oocytes. Brightfield microscopy images demonstrate a darker, more obscured oocyte cytoplasm in lipid-rich horse (A) and pig (B) oocytes, as compared to the more transparent cytoplasm in mouse (C) oocytes.

As the intracytoplasmic lipid content of oocytes varies immensely between species, the precise requirements for individual lipids during oocyte maturation remain somewhat of a mystery (Sturmey & Leese 2003). Notably, those species whose oocytes have a higher lipid content present with a dark, opaque appearance under the microscope, making visualisation and evaluation difficult (Ferguson & Leese 1999, Genicot *et al.* 2005, Hinrichs 2010, Jasensky *et al.* 2016, Morris 2018). Both equine and porcine oocytes are good examples of this (Homa *et al.* 1986, Hinrichs & Williams 1997, Sturmey *et al.* 2009) (Fig. 4), with the immature porcine oocyte typically containing 156 ng of lipid (McEvoy *et al.* 2000), in contrast to mouse oocytes, which typically only contain 3.8 ng of lipid (Amstislavsky *et al.* 2019). A clear relationship exists between lipid content and the cryotolerance of oocytes (Nagashima *et al.* 1995); a high intracellular lipid content causes physical damage, impairing cryopreservation (Nagashima *et al.* 1995). Consistent with mammalian oocytes being some of the hardest cells to cryopreserve (Arav 2014), the efficiency of equine oocyte cryopreservation is limited due to poor maintenance of developmental competence (Galli *et al.* 2007, Ducheyne *et al.* 2019).

Amazingly, physical removal of lipid droplets from mature porcine oocytes, by a process known as delipation did not eliminate the potential for full-term development (Nagashima *et al.* 1995, Seidel 2006) and improved cryopreservation efficiency (Grupen 2014). Given the role of lipids in energy production and as precursors in steroidogenic and eicosanoid pathways, it would be beneficial to know the optimal lipid content required to best support cryotolerance and subsequent developmental competence (de Andrade Melo-Sterza & Poehland 2021).

The reason for lipid variation across different species remains unclear. Although they have different gestation lengths (114 days vs 340 days), the pig and the horse both have strikingly long pre-implantation periods, and it has been proposed that the high lipid content of the oocytes of these species is essential for providing nutrients to the developing conceptus during this period (Sturmey *et al.* 2006). Porcine embryos attach to the maternal uterine epithelial surface after day 13 (Bazer & Roberts 1983, Bazer 2013, Zeng *et al.* 2019). Initial equine embryo fixation occurs around day 16 of pregnancy (Aurich & Budik 2015). It has been suggested that the lipid reservoir within the oocyte in polytocous species may be specifically required to provide energy until placental development (Ambruosi *et al.* 2009). However, given the large lipid content of the oocyte in the monotocous horse, the reasons for this are more likely to be species specific, or due to the phylogenetic link between pigs and horses (Carter & Enders 2004). More recently, it has been suggested that oocyte lipid differences occur due to the variance in diapause length in species that exhibit this phenomenon (Arena *et al.* 2021). For example, the roe deer (*Capreolus capreolus*), which exhibits embryonic diapause lasting up to 5 months (Aitken 1981), has a high level of oocyte lipid content; whereas the rat (*Rattus norvegicus*), with its low lipid content, has only a few days’ diapause (Mantalenakis & Ketchel 1966). Such findings indicate that the amount of lipid within oocytes positively correlates with the duration of their species-specific diapause, which is now believed to occur more widely across a variety of species (Ptak *et al.* 2012). In mammals that do not undergo diapause, including humans, it has been suggested that some mechanisms of diapause have been evolutionarily conserved (Tarín & Cano 1999, Ptak *et al.* 2012). Interestingly, horse embryos are unusually tolerant to recipient uterine asynchrony, meaning that donor embryos can be successfully placed in a recipient uterus that is at a slightly different cycle stage. A day 10 equine embryo may be successfully transferred into a uterus that is as many as 7 days behind (Betteridge *et al.* 1982, Wilsher *et al.* 2010, Gibson *et al.* 2018), while post-fixation, the early pregnancy progresses along a defined time-course (Ginther 1992). With this in mind, it is worth considering oocyte lipid content may have a role in equine embryo survival.

Lipid supplementation, either dietary (*in vivo*) or* in vitro* during IVM, may alter the FA composition of the oocyte (Lapa *et al.* 2011, Warzych *et al.* 2011, Prates *et al.* 2013). However, this supplementation is not always beneficial. When bovine COCs were treated with a combination of FFAs (palmitic, stearic) and the unsaturated FA oleic acid during IVM, genes involved in energy metabolism and oxidative stress were upregulated (Van Hoeck *et al.* 2013). Blastocysts from these oocytes had reduced developmental competence and transcriptional changes, such as higher amounts of the glucose transporter, SLC2A1 (Van Hoeck *et al.* 2011). In equine oocytes matured in medium containing serum, an abundance of SLC2A1 was also found in expanded-COCs such that the latter exhibited superior meiotic competence compared with unexpanded-COCs (González-Fernández *et al.* 2018). An explanation may be that FFA exposure creates an imbalance of the intracellular oxidation-reduction potential, as increased reactive oxygen species (ROS) concentrations are known to upregulate SLC2A1 transcription. Unsaturated fatty acids (UFA), such as linolenic acid, are reported to improve maturation via their ability to impact PG production (Marei *et al.* 2009). However, these too can be detrimental, with high doses of linolenic acid-reducing cumulus expansion and impairing the maturation of bovine oocytes (Marei *et al.* 2009). FA saturation status appears to influence oocyte maturation, with UFAs generally supporting oocyte developmental competence and subsequent embryo development (Dunning *et al.* 2014). Such research suggests that the lipid content of IVM media influences the oocyte–lipid pathway and consequently, the developmental capability of the oocyte.

### Lipids in follicular fluid

One of the roles of follicular fluid is to establish a unique micro-environment to enable oocyte maturation within the ovarian follicles. As such, follicular fluid is composed of a dynamic combination of hormones (FSH, LH and oestrogens), growth factors, peptides, proteins and lipids (Appasamy *et al.* 2008, Chen *et al.* 2016), the concentrations of which alter during follicular growth (Rouillier *et al.* 1996). Steroid hormone production increases and the diffusion distance for gasses inside the follicle also increases, eventually leading to a continuous decrease of oxygen concentration in the follicular fluid (Baddela *et al.* 2018). This delicate balance between oxygen tension and steroid hormone production leads to a homeostatic condition, whereby oxidative stress impacts the production of steroid hormones. If the balance is swayed too far, this can result in the condition of oxidative stress, whereby superoxide radicals form and begin to attack lipids. As a protective mechanism, the oocyte utilises oestrogen, which, in addition to its steroidal action, acts as a major form of antioxidant defence (Appasamy *et al.* 2008). Interestingly, in male reproduction, the impact of ROS and toxic aldehydes, such as 4-hydroxynonenal, is a subject of extensive research (Aitken 1999, Wang *et al.* 2003, Baker & Aitken 2005). When looked at in an IVF setting, once lipid peroxidation levels are increased within the follicular fluid, pregnancy outcomes are negatively affected (Das *et al.* 2006, Chen *et al.* 2016, Borowiecka *et al.* 2012, Cordeiro *et al.* 2018). In addition, following ovulation, follicular fluid plays an important physiological role, with some of the biodynamic fluid washing over both oviductal epithelium and if present, spermatozoa (Bromfield *et al.* 2014, Leemans *et al.* 2015). It is well established in the horse (Lange-Consiglio & Cremonesi 2012, Leemans *et al.* 2015), human (Fetterolf *et al.* 1994, Yao *et al.* 2000), bull (Sostaric *et al.* 2005), hamster (Yanagimachi 1969) and rabbit (Harper 1973) that follicular fluid contains factors capable of activating or capacitating spermatozoa present in the oviduct. However, as the equine dominant follicle typically grows to a diameter of 40–50 mm before eventual rupture, anywhere between 30 and 65 mL of follicular fluid is released at ovulation.

In the horse, proteomic follicular fluid composition has been characterised (Petrucci *et al.* 2014, Spacek & Carnevale 2018, Dutra *et al.* 2019, Fernández-Hernández *et al.* 2020) and biomarkers that are predictive of oocyte fertility have been identified (Fahiminiya *et al.* 2011). Collectively, these investigations have shed light on the effect that season and maternal age have on the follicular fluid proteome, informing the future optimisation of equine IVM conditions. However, investigations into the lipid–protein relationship remain underexplored. A recent study of human follicular fluid found that 11 lipids were in higher abundance in an aged group, indicating that the lipid composition of this fluid alters with age (Cordeiro *et al.* 2018). If the proteomic, ROS and lipidomic compositions of follicular fluid prove to be useful in evaluating oocyte quality when selecting oocytes for assisted reproductive technologies in humans, it is tempting to assume that lipidomic composition may be a useful analytical tool for horses as well.

### Lipids in the oviduct

The oviduct is not merely a passive tube for the passage of the spermatozoa, oocyte and embryo, but is a unique hormonally regulated environment, in which complex dynamics support the early embryo’s survival and development (Lyons *et al.* 2006). The dialogue between the embryo and the mare’s reproductive tract almost certainly begins in the oviduct (Betteridge 2000). Interestingly, all classes of lipids can be found in oviductal secretions, mainly bound to high- and low-density lipoproteins (Ménézo *et al.* 2015). After ovulation, the oocyte is picked up by the oviductal infundibulum and travels over the surface to the oviductal ampulla (Smits 2010). Typically, fertilisation by the spermatozoon occurs at the ampullary–isthmic junction (Leemans *et al.* 2016). In the cow, oviductal fluid from the isthmus has a high cholesterol concentration and a low phospholipid content, resulting in higher cholesterol: phospholipid ratios than in ampullary oviductal fluid (Grippo *et al.* 1994). It is believed that cholesterol provides a stabilising environment for sperm membranes, which bind to the isthmic epithelial cells. The bovine embryo lingers in the oviduct for only three days (Hafez & Hafez 2016) and the porcine embryo remains in the oviduct for only two days (Dziuk 1985). However, in the mare, the developing embryo remains in the oviduct as its cells proliferate and differentiate for 5 days following fertilisation (Betteridge *et al.* 1982, Freeman *et al.* 1991, 1992), before passing through the utero-tubal junction into the uterus at approximately day 6 (Battut *et al.* 1997).

There are three components that contribute to successful oviductal transport: ciliary beat, muscular contractility and tubal secretions (Ezzati *et al.* 2014). Although all three need to cooperate for the successful transport of the embryo, the lipid content of the secretions is of particular interest. In the mare, as with follicular fluid, the amount of oviductal fluid secreted is comparably large. When oviductal cannulas were used to measure secretions, the mean daily secretion rate in pony mares ranged from 0.8 to 3.5 mL during the luteal phase and from 3.2 to 6.4 mL during the oestrus (Engle *et al.* 1970). Although oviductal catheterisation provides useful insights, the technique undoubtedly induces an inflammatory response within the tissue and as such can lead to misinformation regarding secretion composition (Saint-Dizier *et al.* 2019). Interestingly, porcine embryo cleavage and blastocyst formation rates were significantly greater when oocytes were treated with raw oviductal fluid (Romar *et al.* 2001). Such embryos expressed a clear anti-apoptotic gene expression profile, which suggests that the oviductal secretions played a protective role against apoptosis (Lloyd *et al.* 2009). These findings reinforce the fact that the oviduct provides not only a venue for fertilisation and early embryo development but also an environment of biochemical support.

Given the high abundance and diversity of lipids in oviductal secretions, speculations about the role of lipids in embryonic development can be made (Saint-Dizier *et al.* 2019). Studies involving equine oviductal explants often experience difficulties in keeping cultures viable long enough to observe normal function and normal gene expression (Critoph & Dennis 1977, Nelis *et al.* 2014). However, it has been noted that when mature oocytes and spermatozoa are placed surgically in the mare oviduct, fertilisation occurs (Carnevale *et al.* 2000, Scott *et al.* 2001). In humans, the underlying oviductal mechanisms governing epithelial homeostasis remain unclear (Ghosh *et al.* 2017), but the micro-environment within the oviduct appears to be conserved between species. The cascade of events that preserve oviductal homeostasis during early fertilisation has some species variations (Avilés *et al.* 2010). Oviductal secretions contain a diversity of lipids, including cholesterol, triglycerides and FAs (Jordaens *et al.* 2017), but the secretions also contain l-carnitine, which is required for the beta-oxidation of these lipids by the mitochondria (Ménézo *et al.* 2015). Additionally, a mixture of glycerophospholipids and sphingolipids, which are membrane lipids implicated in many cell signalling pathways, was recently identified in bovine oviductal tissues and secretions (Banliat *et al.* 2019). In the mouse, the embryo-derived phospholipid PAF appears to cause an acute consumption of platelets in the microvasculature of the oviduct (O’Neill 1985). This consumption can also be observed systemically in some species during early pregnancy with resulting thrombocytopenia. A diagnosis of thrombocytopenia is characterised by abnormally low levels of platelets in the circulating blood. Such events illustrate that biological incidents that occur within the oviduct can stimulate a systemic response. In women, thrombocytopenia occurs in some cases and thrombocytosis (increased platelets) in others (Yeung *et al.* 1992). Localised oviductal platelet consumption has also been seen in both the bovine (Kojima *et al.* 1996*a*) and rabbit (Kojima *et al.* 1996*b*), but has not yet been explored in the horse.

However, interestingly fertilised equine oocytes can ‘overtake’ unfertilised ova within the oviduct (Betteridge 2000). This phenomenon occurs in many species, it was first identified in the horse (van Niekerk & Gerneke 1966). The ability of the oviduct to differentiate between unfertilized oocytes and developing embryos is based on the fact that only the latter secrete PGE_2_. Indeed, when an embryo reaches the compact morula stage of development on day 5 it begins to secrete appreciable quantities of this hormone (Weber *et al.* 1991), which acts locally to relax the circular smooth muscle fibres in the oviduct wall causing the ampullary–isthmus sphincter to open, and thereby allowing the embryo to enter the uterus. It has been proposed that the embryo-derived PGE_2_ is responsible for increased oviductal contractions and escalation in ciliary beat (Weber *et al.* 1991, 1995, Robinson *et al.* 2000). PGE_2_ binds specifically to the horse oviduct (Weber *et al.* 1992); in fact, the PGE_2_ receptors EP2 and EP4 are strongly expressed in both the isthmic and ampullar epithelium of the oviduct (Ball *et al.* 2013). Moreover, these receptors are upregulated once ovulation occurs, regardless of whether fertilisation has occurred or not. As such, a more defined understanding of the lipidomic content of the oviduct structure and its secretions may enhance our knowledge in the field of equine IVF (van Niekerk & Gerneke 1966).

### The lipidome of uterine fluid ‘histotrophe’

Once the conceptus has entered the uterus, the histotrophe provides the main interface for communication between conceptus and uterus prior to implantation around day 40 post-ovulation (Zavy *et al.* 1982, Bazer & Roberts 1983, Stewart *et al.* 2000). The equine histotrophe is a dynamic fluid consisting of lipids, hormones, growth factors, cytokines and proteins, the latter of which have been the focus of several recent investigations (Suire *et al.* 2001, Stewart *et al.* 2000, Fahiminiya *et al.* 2011, Smits *et al.* 2017, Swegen *et al.* 2017, Lawson *et al.* 2018). The predominant protein of the equine uterine histotrophe is uterocalin (P19), with a large amount of P19 being present during the first 23 days of pregnancy (Stewart *et al.* 2000, Suire *et al.* 2001). P19 is a member of the lipocalin family of proteins which are lipid transporters (Flower 1996). In the horse, P19 binds a range of biologically important lipids, including polyunsaturated FAs, and is believed to be involved in providing the appropriate pre-attachment environment for the equine embryo, functioning as a carrier for essential lipids and amino acids (Suire *et al.* 2001). Histotrophe provides a rich source of information regarding the cellular processes underlying embryo-maternal interactions. Currently, very little is known about the histotrophe lipidome, and as such, there is a substantial amount of work to be done to identify factors responsible for embryo support prior to implantation in the horse.

### The uterus

Once in the uterus, the conceptus propels itself via the production of PGs, which cause myometrial contractions (Stout & Allen 2001b), though the endometrial lining of the uterus does not become receptive to the embryo for another 10 days. This prolonged migration is unique to the horse, and it has been suggested that its purpose is to deliver a secretory factor across the endometrium for MRP (McDowell *et al.* 1988). As previously discussed, endometrium morphological changes must occur in order to be receptive to the embryo, and it is likely that lipids play a role in these changes (Hall 1975, Klein 2016).

The importance of lipids during implantation has been demonstrated (Wang & Dey 2005, Sordelli *et al.* 2012, Vilella *et al.* 2013), with each species presenting a unique variation in the biochemical endometrial cascade. Understanding the intricacies of other species has paved the way for research on the horse. In the pig, sheep, cow, roe deer, ferret, cat, rabbit and horse, oestrogens are produced by the pre-implantation embryo (Gadsby *et al.* 1980, Heap *et al.* 1982). It has been shown in the pig, cow and ewe (Chenault 1980, Ford *et al.* 1982, Reynolds *et al.* 1984) that these oestrogens stimulate increased uterine blood flow in order to support the pregnancy. In hamsters and mice, implantation is related to a PGE_2_ increase through the co-expression of both prostaglandin E synthase 2 and COX2 at the implantation site (Wang & Dey 2006, Vilella *et al.* 2013), and in the mouse, a lack of either PLA2 or COX2 leads to an absence of PG synthesis and subsequent implantation defects. In humans, the enzymes responsible for PG production, PLA2 and COX2, increase in both the lumen and the stroma throughout the receptive period (Fortier *et al.* 2008) and alteration in the PG pathway directly affects the process of implantation. An investigation into the endometrium of day 13.5 pregnant mares revealed a downregulation of oestrogen receptor 1 (ESR1) and an upregulation of an amino acid transporter (solute carrier family 36 member 2; SLC36A2) (Klein *et al.* 2010), presumably to provide adequate energy to the developing embryo. However, this is in contrast to other studies that showed no difference in ESR1 expression between day 5 and day 15 of pregnancy (McDowell *et al.* 1999) and indeed lower levels of ESR1 and progesterone receptors between day 11 and day 20 of pregnancy (Hartt *et al.* 2005). Despite the lack of consensus regarding the timing of change in ESR1 levels, it is assumed that lipids, specifically PG and oestrogen, contribute to establishing the micro-environment required for equine embryo implantation and hence why the lipidomic profile of the uterus is of particular interest.

### The embryo

There are several features of the equine embryo, including the presence of an encasing acellular glycoprotein coat, a long pre-implantation period and a highly mobile, spherical conceptus, which make the species very unique (Short 1969). While much is known about the metabolism of exogenous nutrients such as glucose, lactate and pyruvate, the role of endogenous energy sources including lipids, has been largely under-investigated (Sturmey *et al.* 2009). At the pre-implantation stage of development, embryos require the biosynthesis of lipids in order to be viable, particularly for energy metabolism, membrane construction and signalling events involved in gene activation (Dutta-Roy 1997). This self-reliance indicates that equine embryos autotrophically produce their own lipid supply, contributing directly to the steroid environment of the intrauterine lumen (Sharp 2000). It is well established that the conceptus secretes PGF_2α_ along with other PGs (Stout & Allen 2002). Steroidogenesis in the equine embryos appears to begin as early as day 6 (Paulo & Tischner 1985), with detectable secretions of oestrogen observed as early as day 7 (Raeside *et al.* 2004), and progesterone detected on day 8 in blastocysts produced* in vitro*. Although steroid hormone production takes place in very early pre-implantation horse embryos, it is the E2 production by the early conceptus that is considered significant to the establishment of pregnancy (Zavy *et al.* 1984, Choi *et al.* 1997, Raeside *et al.* 2004). Of interest, apolipoproteins have been previously reported in transcriptome studies of the equine embryo and endometrium (Klein & Troedsson 2011*b*, Swegen *et al.* 2017). Most commonly their presence is attributed to the nutritional demands of the embryo and the transport of lipids to support these demands. Human studies revealed lower levels of secreted APOA1 were predictive of a successful human pregnancy (Nyalwidhe *et al.* 2013), suggesting that the embryo’s capacity to bind and/or internalise APOA1 might be representative of its competence. Taken together, it cannot be assumed that any of the hormones and/or lipids mentioned are working in isolation but are working as part of a complex interplay of interactions. Other embryo-produced mediators, such as the phospholipid PAF, may exert a stimulatory effect (O’Neill 2005), and eicosanoid precursors such as AA and docosahexaenoic acid, which are essential constituents of the membrane lipids in other species (Dutta-Roy 1997), could play crucial roles in equine embryonic development.

A recent study, which investigated the embryonic secretome of day 9 and day 10 equine embryos found an increase in the incidence of lipid, glycolipid, phospholipid, cholesterol and lipoprotein-associated biological processes (Swegen *et al.* 2017). Such findings implicate the role of lipids and protein–lipid complexes in supporting the early equine embryo, particularly at the pre-implantation stage. As such, the production of lipid appears to be necessary to meet the energy needs of the growing pre-implantation embryo. The timing and amount of this lipid fraction appears to be species specific.

### Equine maternal recognition of pregnancy

**Figure 5:**
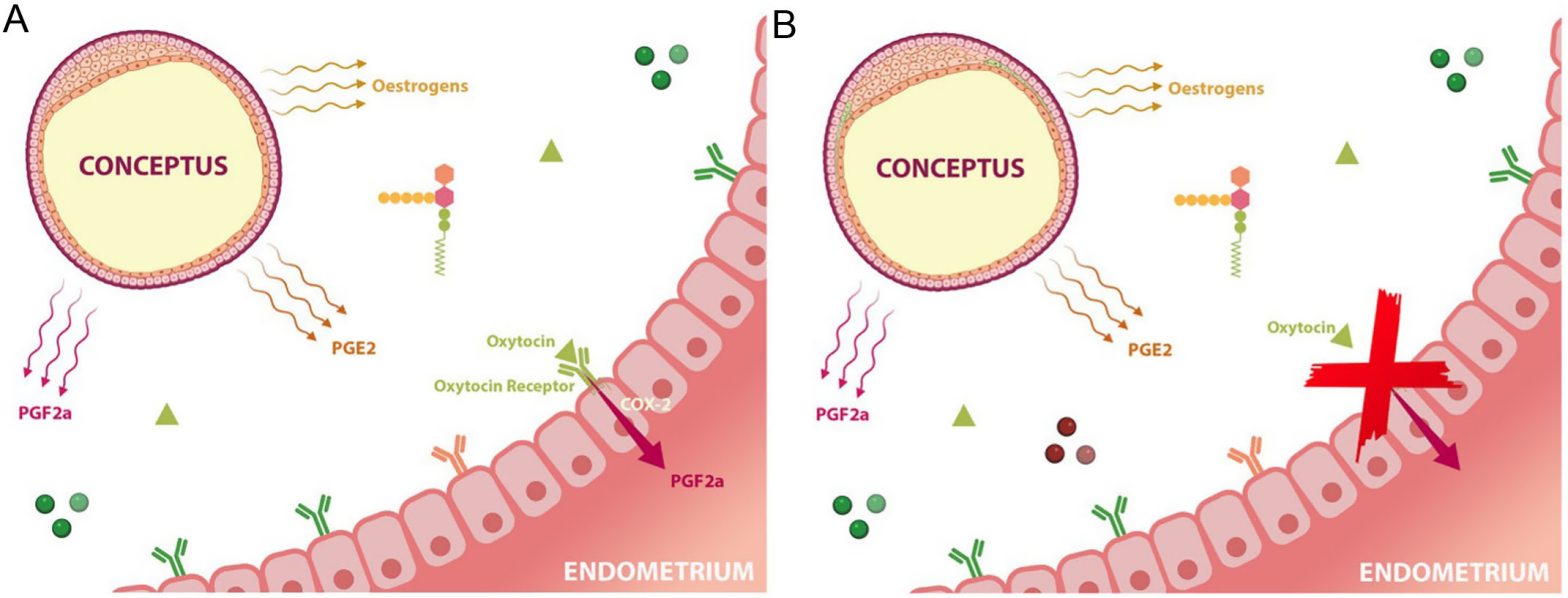
A representation of the equine conceptus within the uterine environment around the time of MRP, on day 12 (A) and day 14 (B) post-ovulation. The conceptus propels itself through the uterus, secreting PGs and E2 into the lumen. Due to downregulation of oxytocin receptors and of COX2 in the endometrial epithelium, oxytocin is unable to contribute to the stimulation of endometrial synthesis of PGF_2α_ and the CL is maintained. CL, corpus luteum; MRP, maternal recognition of pregnancy; PGs, prostaglandins.

The MRP factor is yet to be identified in the horse. One hallmark of the equine MRP is the conceptus initiated down-regulation of PGF_2α_ in the endometrium, thereby preventing CL lysis (de Ruijter-Villani *et al.* 2015). This anti-luteolytic signal enables the continued production of ovarian progesterone (Sharp *et al.* 1997) and pregnancy maintenance. The downregulation of PGF_2α_ is due to the attenuation of intrauterine oxytocin receptor expression, and hence COX2 at a post-transcriptional level (Klein 2015) (Fig. 5). The cascade of events that precede MRP, including ovulation, fertilisation, oviductal transport and the 10 day sojourn of the conceptus within the uterus, can all be better understood with a clearer appreciation of the lipid–protein interactions. Although MRP is not a key focus of this review, equine conception and pregnancy cannot be discussed without acknowledging the significance of this yet still unidentified biomarker of MRP in the horse, which may be revealed through lipidomic studies.

## Lipidomic challenges

There are always challenges that arise with the dawn of any emerging technology, which has been the case with lipidomics. In the field of lipidomics, there are large disparities in methodologies and technologies between studies, which have resulted in inconsistencies in published results (Liebisch *et al.* 2019). The field has advanced more rapidly than universally accepted protocols and standardisation of the techniques could occur. A good example of this in the context of the equid is a recent study in which non-targeted lipidomics were used to detect O-acyl-omega-hydroxy-FA (OAHFA) in both the head of the equine spermatozoon (Wood *et al.* 2016) and in equine amniotic fluid (Wood *et al.* 2018). OAHFA are a recently discovered family of lipids (Hancock *et al.* 2018) that to date have only otherwise been found in human skin (Hirabayashi *et al.* 2017) and the meibomian gland secretions of the eyelids (Butovich *et al.* 2009, Butovich *et al.* 2012). A lack of standardisation of sample preparation strategies and the use of shotgun mass spectrometry to detect OAHFAs in equine samples caused some debate, with a suggestion that their presence was an artefact (Chen *et al.* 2010). Such quandaries have been common, but as standardisation improves, such debates should fade. Furthermore, like much of the metabolomic research carried out in equine species, horse-specific data within the field is limited, and there are only a few studies investigating equine-specific lipidomics. However, as protocols for lipid extraction, processing, identification and characterisation of lipids become standardised, more opportunities will arise to better understand the roles of lipids in equine reproductive biology.

## Conclusions

It is well known that lipid metabolism represents a systematic interaction of gene, protein, metabolite, lipid and enzyme (Dutta-Roy 1997). Apart from the roles of lipid-based steroid hormones in equine reproduction, not much is known about the roles of the other classes of lipids. It is established that localised lipid metabolism greatly changes during early fertilisation and pregnancy (Adank *et al.* 2020), but it is an under-investigated field of research in equine species. Understanding the roles of lipids during the preconception period and during early pregnancy, may provide a valuable avenue to identify biomarkers of both fertility and early pregnancy. Furthermore, the lipidomic profiling of both follicular fluid and the oviductal secretome will provide valuable insights into the pathways and mechanisms surrounding fertilisation. The effects of various follicular fluid lipids on oocytes, spermatozoa and oviductal epithelial cells will be pivotal in increasing our understanding of the biochemical cascade of events leading to fertilisation. This will potentially advance equine ART and improve pregnancy outcomes and foaling rates. Furthermore, these developments could bring the industry closer to making conventional IVF in horses possible, which would undoubtedly be a great scientific achievement. In-depth lipidomic processes are just starting to be explored. Despite standard protocols not yet being fully established, recent innovations in lipidomics and the elucidation of some of the complex pathways involved with the synthesis of lipids has promising potential for future research. The study of lipids, the use of lipidomics and the upskilling of equine researchers with lipidomic technologies, will undoubtedly progress the field of equine reproductive research, with the potential to solve the reproductive quandaries involved with equine fertilisation and embryo–maternal communication, and thus improve clinical practice.

## Declaration of interest

The authors declare that there is no conflict of interest that could be perceived as prejudicing the impartiality of this review.

## Funding

This work was funded by the Australian Research Council http://dx.doi.org/10.13039/501100000923 (LP160100824).

## Author contribution statement

Edwina F Lawson contributed to conceptualisation, wrote the manuscript and created the figures. Zamira Gibb contributed to conceptualisation, manuscript writing, editing and provided supervision. Christopher G Grupen contributed to manuscript writing, review and editing. Mark A Baker contributed to manuscript visualisation and editing. R John Aitken provided supervision and contributed to manuscript review and editing. Aleona Swegen contributed to manuscript review and editing. Charley-Lea Pollard contributed to the figures.
